# Synergy effect of peroxidase enzymes and Fenton reactions greatly increase the anaerobic oxidation of soil organic matter

**DOI:** 10.1038/s41598-020-67953-z

**Published:** 2020-07-09

**Authors:** Carolina Merino, Yakov Kuzyakov, Karina Godoy, Pablo Cornejo, Francisco Matus

**Affiliations:** 1 Laboratory of Conservation and Dynamic of Volcanic Soils, Department of Chemical Sciences and Natural Resources, Universidad de La Frontera, Temuco, Chile; 2 Center of Plant, Soil Interaction and Natural Resources Biotechnology. Scientific and Technological Bioresource Nucleus (BIOREN), Universidad de La Frontera, Temuco, Chile; 3 Network for Extreme Environmental Research (NEXER) Universidad de La Frontera, Temuco, Chile; 4 Soil Science of Temperate Ecosystems, Büsgen Institute, Georg-August-Universität Göttingen, Göttingen, Germany; 5 Agro-Technological Institute, RUDN University, 117198 Moscow, Russia; 6 Departamento de Ciencias Químicas Y Recursos Naturales, Universidad de La Frontera, Temuco, Chile

**Keywords:** Biogeochemistry, Environmental sciences

## Abstract

In temperate rainforest soils of southern Chile (38 °S), there are high rates of soil organic carbon (SOC) mineralization under oxygen (O_2_) limitation. We study the combined effects of Fenton reactions and the activity of two enzymes manganese peroxidase (MnP) and lignin peroxidase (LiP), which was hypothesised potentiate SOC mineralization under anoxic conditions leading to carbon dioxide (CO_2_) release. Both mechanisms produce free radicals when competing for SOC oxidation in the absence of microorganisms. We quantify the CO_2_ release by induced Fenton reactions in combination with MnP and LiP under aerobic and anaerobic conditions (20 °C, 36 h) in temperate rainforest soils. CO_2_ levels released by Fenton reactions and enzyme activity were eight times higher than those released by Fenton reaction and peroxidase enzymes in individual treatment. Approximately 31% of the CO_2_ released under aerobic soil incubation was found to be abiotic (sterilized), while 69% was biotic (non-sterilized soils), and respective values of 17% and 83% were recorded under anaerobic conditions. The relative fluorescence intensity clearly shows ·OH radicals production from Fenton reactions. In conclusion, levels of MnP and LiP coupled with Fenton reactions strongly increase SOC mineralization under long periods of O_2_ limitation in temperate rainforest soils.

## Introduction

Our current understanding of iron (Fe) as a driver of abiotic CO_2_ release comes almost exclusively from tropical soils with high Fe content^1,2^. The effects of warm temperatures, high rainfall (> 3,000 mm year^−1^), topography, and vegetation on rapid soil organic matter (SOM) decomposition have been studied intensively in tropical soils^3–5^. Despite the importance of these studies, the contributions of combined mechanisms, Fenton reactions and the enzyme activity of manganese peroxidase (MnP) and lignin peroxidase (LiP) to CO_2_ release from temperate rainforest soils have never been explored. MnP and LiP catalyse a variety of oxidative reactions^6^ and can persist in anaerobic soil microsites^7,8^. Fenton reactions in combination with the activity of these enzymes can potentiate SOC mineralization due to the production of free radicals (strong oxidants of SOM) competing for soil organic matter (SOM) oxidation under anoxic conditions. This perspective challenges the traditional assumption that the soils of humid temperate rainforests, especially under high levels of precipitation, mineralize slowly due to a high proportion of anaerobic soils microsites. More and more evidence of abiotic mechanisms involving Fe redox processes that can lead to substantial C losses has been reported^3,4^. In anaerobic microsites, Fe transfers electrons during redox reactions, which can serve as an energy source to produce free radicals (e.g., hydroxyl radicals) for the abiotic oxidation of soil organic carbon (SOC)^1,9,10^. Hydroxyl radicals are strong non-selective oxidants that can attack similar to the lignin structures of SOM^6,11^. Thus, LiP and MnP combined with Fenton chemistry may accelerate C turnover in soils with high Fe content.

In Chile, most humid temperate rainforest (*Nothofagus *spp., *Araucaria araucana*) stands in the Andes and Coastal Cordillera receive up to 8,000 mm year^−1^^12^. Such conditions create temporary O_2_ limitation that controls C dynamics^13–15^. Hydrogen peroxide is produced in aerobic microsites via water photolysis in the presence of light^16^ and diffuses into anaerobic microsites in soil. Fenton reactions and particularly LiP yield ·OH, reacts with recalcitrant lignin-like and other components of SOM.

We hypothesize that Fenton reactions in combination with the activities of ligninolytic enzymes form a complementary mechanism of CO_2_ release, enhancing the oxidation of SOC under O_2_ limitation conditions in the absence of microorganisms. The objective of the present study was to quantify C release by Fenton reactions at different ratios of H_2_O_2_:Fe(II) in combination with MiP and LiP in temperate rainforest soils. To broaden the scope of our study, we evaluated three soil types differing in Fe content, pH, and parent material origin.

## Results

### Abiotic and biotic incubation

**Figure 1:**
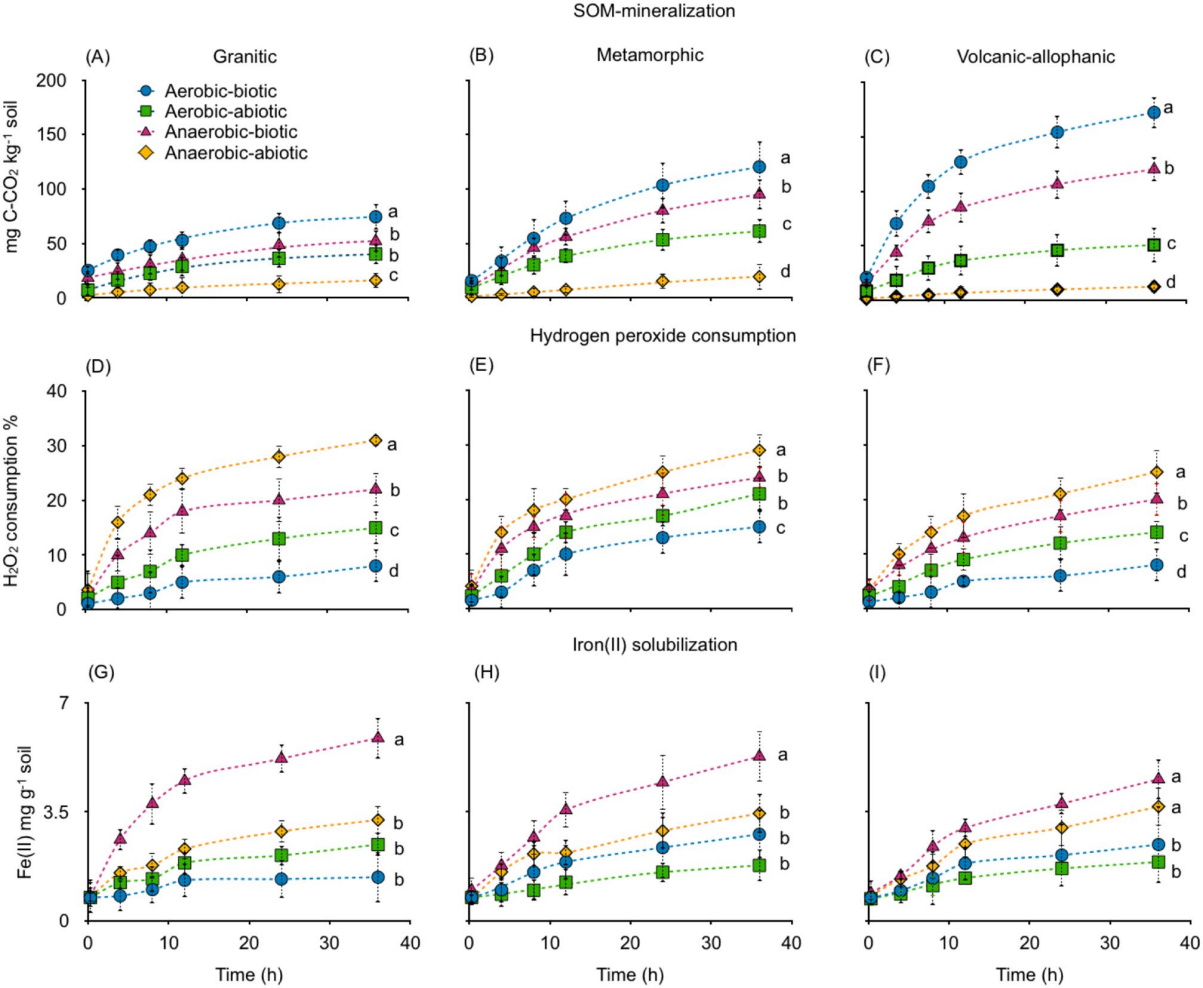
(**A**–**C**) CO_2_ evolved from aerobic and anaerobic sterilized (abiotic) and non-sterilized (biotic) soils derived from granitic, metamorphic and volcanic-allophanic parent materials incubated at 20 °C for 36 h. (**D**–**F**) H_2_O_2_ consumption and (**G**–**I**) Fe(II)-HCl solubilization. Different letters shown in each panel indicate significant differences (*p* < 0.05).

**Figure 2:**
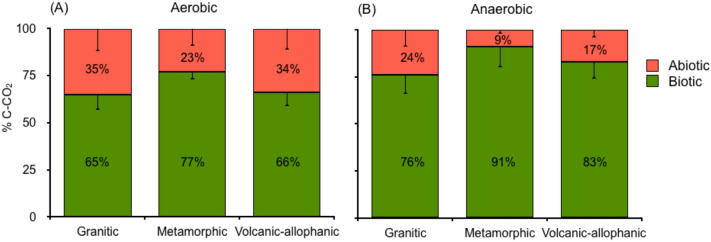
Total CO_2_ proportion of sterilized (abiotic) and non-sterilized (biotic) soils derived from granitic, metamorphic and volcanic-allophanic parent martials incubated at 20 °C for 36 h under (**A**) aerobic and (**B**) anaerobic conditions. Different letters shown in each panel indicate significant differences (*p* < 0.05).

After 36 h of incubation, similar patterns of CO_2_ release were recorded in all soils. The highest values were found during the aerobic incubation of non-sterilized soils (biotic). Volcanic-allophanic soil showed the highest levels of CO_2_ (172 mg C kg^−1^) followed by Metamorphic and Granitic soils (Fig. 1A–C, Table S1, Supplementary Information). The lowest levels of C mineralization were recorded in sterilized soils (abiotic) under anaerobic incubation. These results are consistent with the consumption of hydrogen peroxide; Anaerobic abiotic, where Fenton reactions are assumed to occur, showed the highest levels of H_2_O_2_ consumption while aerobic biotic incubation showed the lowest (Fig. 1D–F). However, Fenton reactions did not show the maximum Fe(II)-HCl extractable, it was higher for Anaerobic biotic (Fig. 1G–I). CO_2_ from Aerobic abiotic and abiotic presented similar levels of H_2_O_2_ consumptions and iron solubilisation. On average, 31% of all CO_2_ released via aerobic incubation was purely abiotic and 69% was released through microbial respiration (Fig. 2A) while under anaerobic incubation, 17% was abiotic and 83% was released through microbial respiration (Fig. 2B).

The CO_2_ from Anaerobic biotic and Aerobic abiotic incubations presented intermediate values and similar pattern of H_2_O_2_ consumption. Fe(II)-HCl extractable (Fig. 2G–I) were higher for Anaerobic biotic followed by Anaerobic abiotic. Iron from the last incubation was not different than others treatments. All these results were expected and are discussed in the corresponding section.

**Figure 3:**
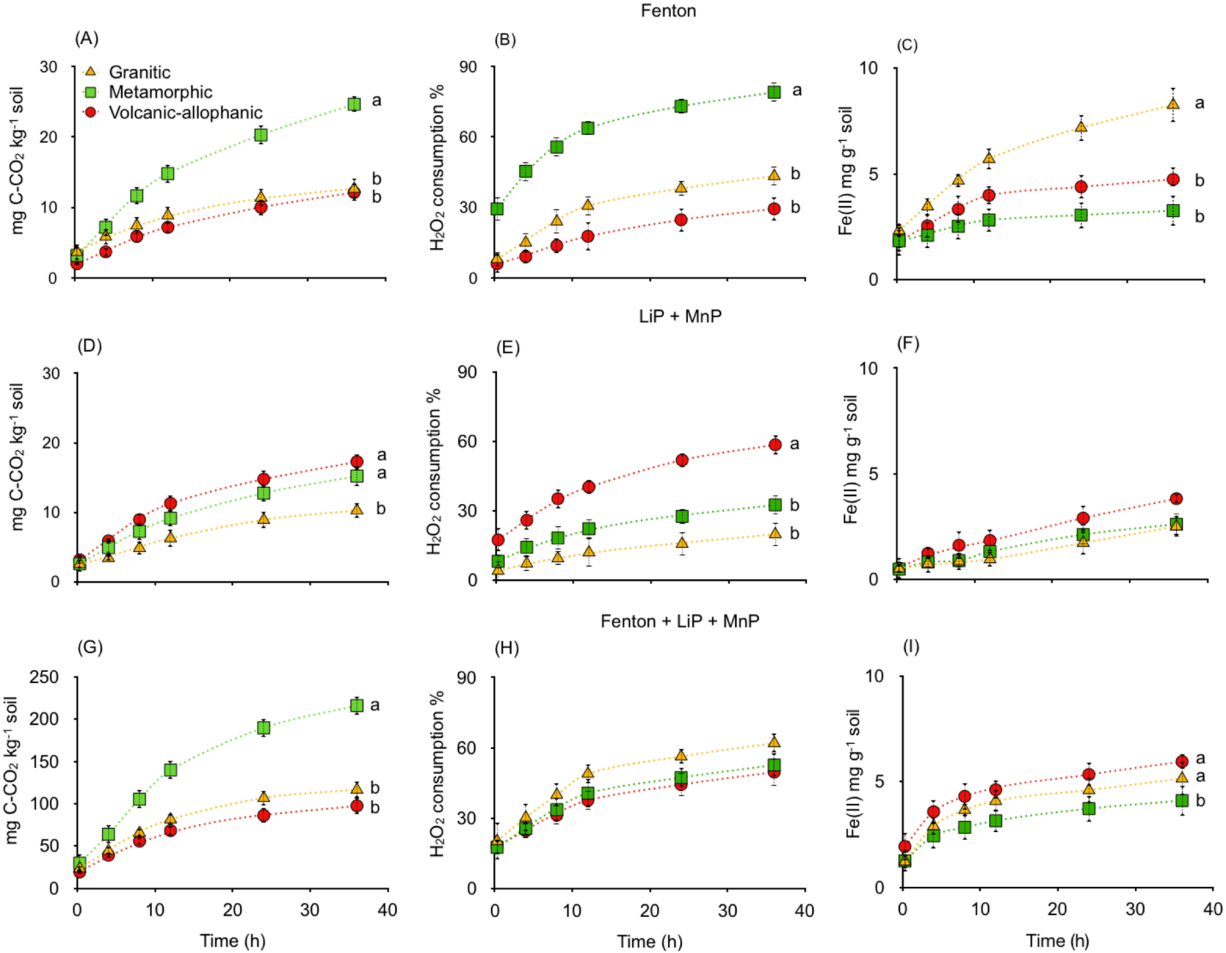
(**A**–**C**) Fenton reactions, (**D**–**F**) activity of enzymes lignin peroxidase (LiP) and manganese peroxidase (MnP) and (G-I) Fenton + LiP + MiP showing the CO_2_ evolution, H_2_O_2_ consumption and Fe(II)-HCl solubilization of soils derived from granitic, metamorphic and volcanic-allophanic parent materials. Different letters shown in each panel indicate significant differences (*p* < 0.05).

Summing up the total CO_2_ released from aerobic incubation, 31% was purely abiotic and 69% corresponded to biotic microbial respiration. While in anaerobic incubation, these values were 17% and 83%, respectively (Fig. 3).

### Induced Fenton reactions and enzyme lignin peroxidase and manganese peroxidase

CO_2_ released by Fenton reactions induced by the addition of various H_2_O_2_:Fe(II) ratios presented significant differences (*p* = 0.03) (Fig. 3, Table S2, Supplementary Information). The optimum Fenton reaction was found to present a H_2_O_2_:Fe(II) ratio of 10:1 for Metamorphic soil, showing the highest CO_2_ value (25 mg kg^−1^) (Fig. 3A). These results are in line with the consumption of hydrogen peroxides. H_2_O_2_ is used in Fenton reactions as a substrate together with Fe(II) (Fig. 3B) and both comparaed with MnP and LiP enzymes, were generally lower than those values produced by Fenton reactions (Fig. 3D–F, Table S2, Supplementary Information). However, when MnP and LiP were combined with H_2_O_2_ and Fe(II) to induce Fenton reactions, there was 4–10 times more CO_2_ (Fig. 3G–I) than any other treatment.

### Enzymatic activity

**Figure 4:**
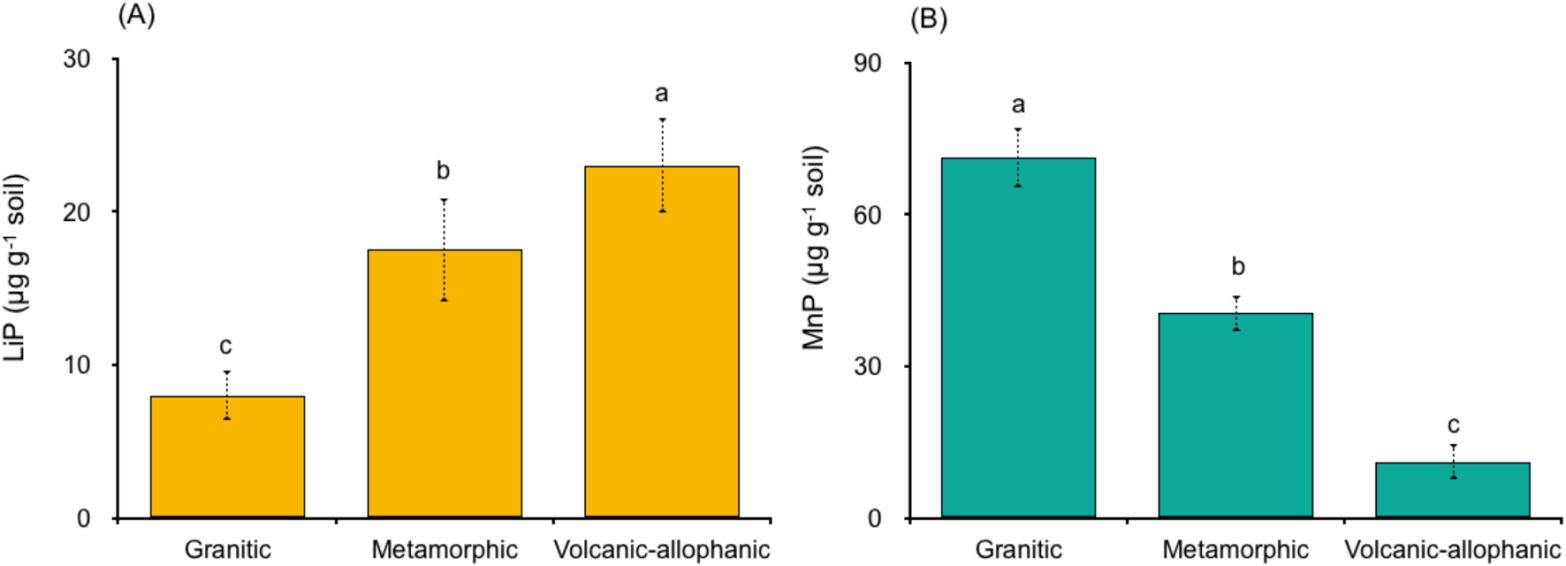
(**A**) Enzyme activity of lignin peroxidase (LiP) and (**B**) manganese peroxidase (MnP) in sterilized soils derived from granitic, metamorphic and volcanic-allophanic soils incubated at 20 °C for 36 h under anaerobic conditions. Note the y-axes of different scales. Bars with different letters shown in each panel indicate significant differences (*p* < 0.05).

The enzyme concentration (soluble crude protein) obtained from the cultured filtrates of white rotted fungi grown after five days of incubation was 614 μg ml^−1^. LiP varied between 5 and 25 μg g^−1^, and MnP ranged from 6 to 75 μg g^−1^ (Fig. 4). Enzyme activity differed across the soils. LiP steadily increased in the following order: Granitic < Metamorphic < Volcanic-allophanic (Fig. 4A). For, MnP the opposite was found (Fig. 4B).

### Relationships between variables measured

**Figure 5:**
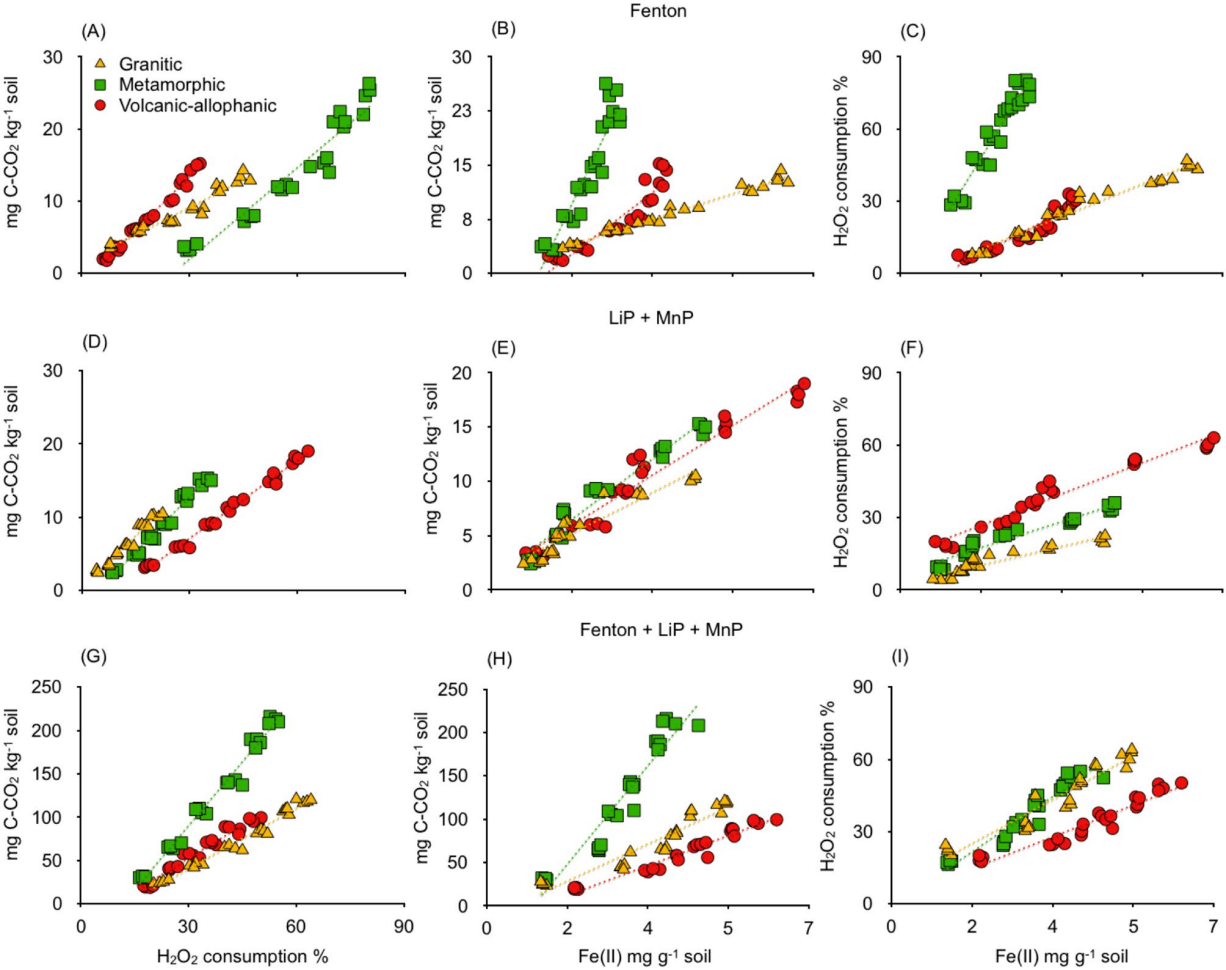
(**A**–**C**) Fenton reaction, (**D**–**F**) activity of enzymes lignin peroxidase (LiP) and manganese peroxidase (MnP) and (**G**–**I**) Fenton + LiP + MiP showing relationships between CO_2_ and H_2_O_2_ consumption, CO_2_ and Fe(II)-HCl and H_2_O_2_ and Fe(II)-HCl for soils derived from granitic, metamorphic and volcanic-allophanic parent materials. All relationships are significant at p < 0.01 (Table 3S, Supplementary Information).

A positive and significant relationship was found between CO_2_ and H_2_O_2_ and between CO_2_ and Fe(II)-HCl. Similar results were obtained for the correlation between and H_2_O_2_ and Fe(II)-HCl (*p* < 0.01, R^2^ > 0.86, Fig. 5) (Table S3, Supplementary Information). The regression slope for the Fenton + MnP + LiP treated soils was measured as eight times higher than those of the Fenton reaction and MnP + LiP experiment alone (Fig. 5).

### Iron balance

After 36 h of incubation, the solubilization of Fe(II)-HCl averaged 5 g kg^−1^. At the beginning of the incubation period, the average amount of Fe(II)-HCl extractable was 4.05 g kg^−1^ in the soil (Table 1). When we add the amount of Fe(II) (1.29 g kg^−1^) to the initial amount of Fe(II)-HCl, the total quantity of Fe(II)-HCl is 5.34 g kg^−1^, which is close to the initial average of iron of soil. Thus, we assume that Fenton reaction reused Fe(III) produced as ferric oxyhydroxides in the range of pH found in the studied soils (3.6–5.1).

**Table 1:** Iron content (g kg^−1^ soil) of studied soils.

Analysis	Granitic	Methamorphic	Volcanic–allophanic
Total Fe^a^	10.4 ± 0.3	11.8 ± 0.5	8.4 ± 0.5
Fe^2+^	2.3 ± 0.5	5.8 ± 0.4	3.2 ± 0.2
Fe^3+^	8.1 ± 0.3	6.0 ± 0.5	5.2 ± 0.3
Fe_d_^b^	8.2 ± 0.1	3.1 ± 0.0	1.2 ± 0.1
Fe_o_^c^	6.1 ± 0.2	2.3 ± 0.3	1.4 ± 0.1
Fe_p_^d^	0.7 ± 0.02	0.9 ± 0.04	0.7 ± 0.1
Fe_c_^e^	2.0 ± 0.2	3.7 ± 0.2	3.8 ± 0.2

^a^Sum of Fe^2+^ and Fe^3+^

^b^Dithionite extractable iron.

^c^Oxalate extractable iron.

^d^Pyrophosphate extractable iron.

^e^Crystalline iron Fe^3+^ minus Fe_o_.

### Fluorescence intensity

**Figure 6:**
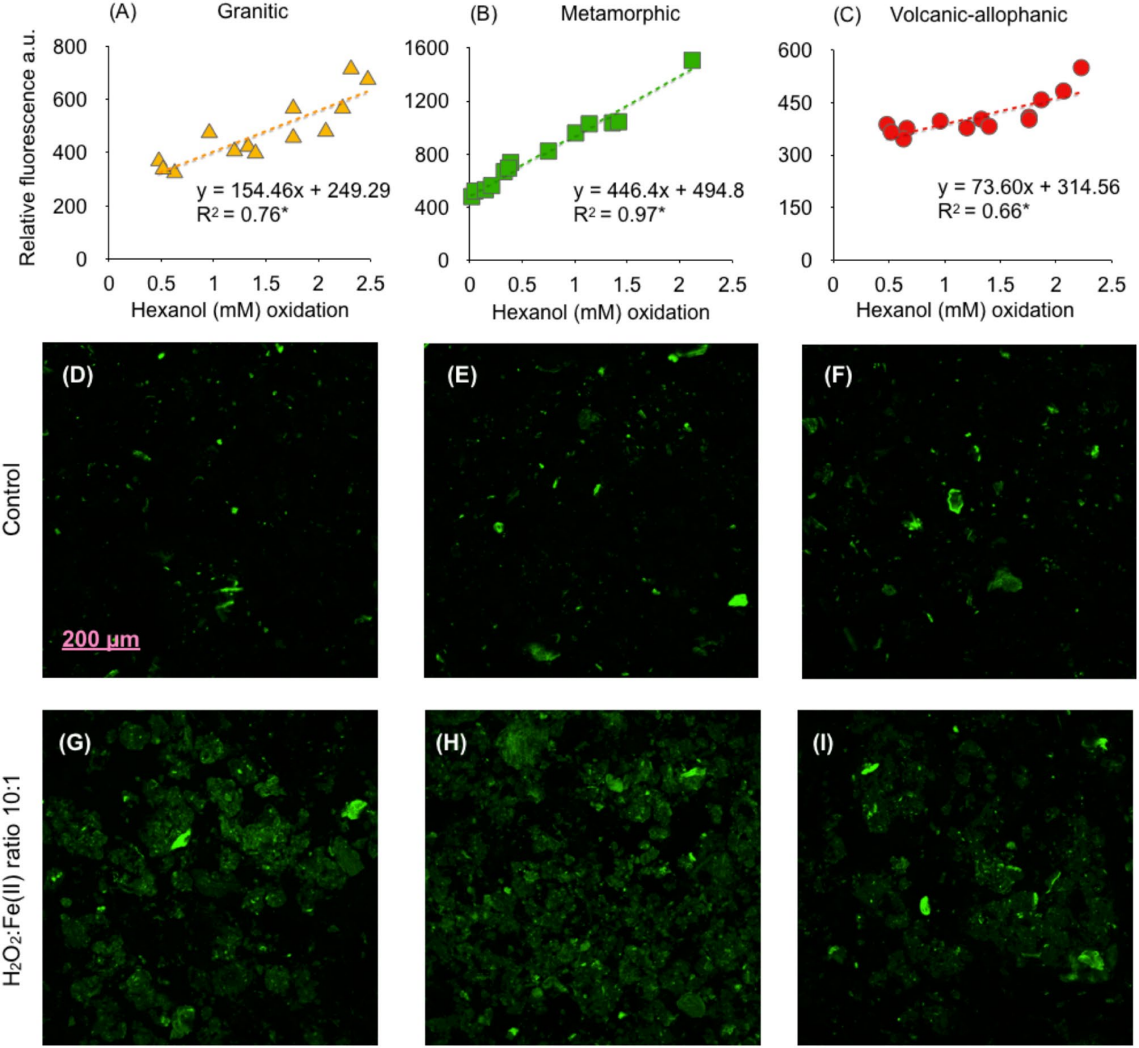
(**A**–**C**) Relationship between hexanol oxidation and relative fluorescence from sterilized soils derived from granitic, metamorphic, and volcanic-allophanic parent materials incubated at 20 °C for 36 h under anaerobic conditions. (**D**–**F**) Confocal laser scanning fluorescence image of the control soil and (**G**–**I**) free hydroxyl radicals at a 10:1 H_2_O_2_:Fe(II) ratio.

**Figure 7:**
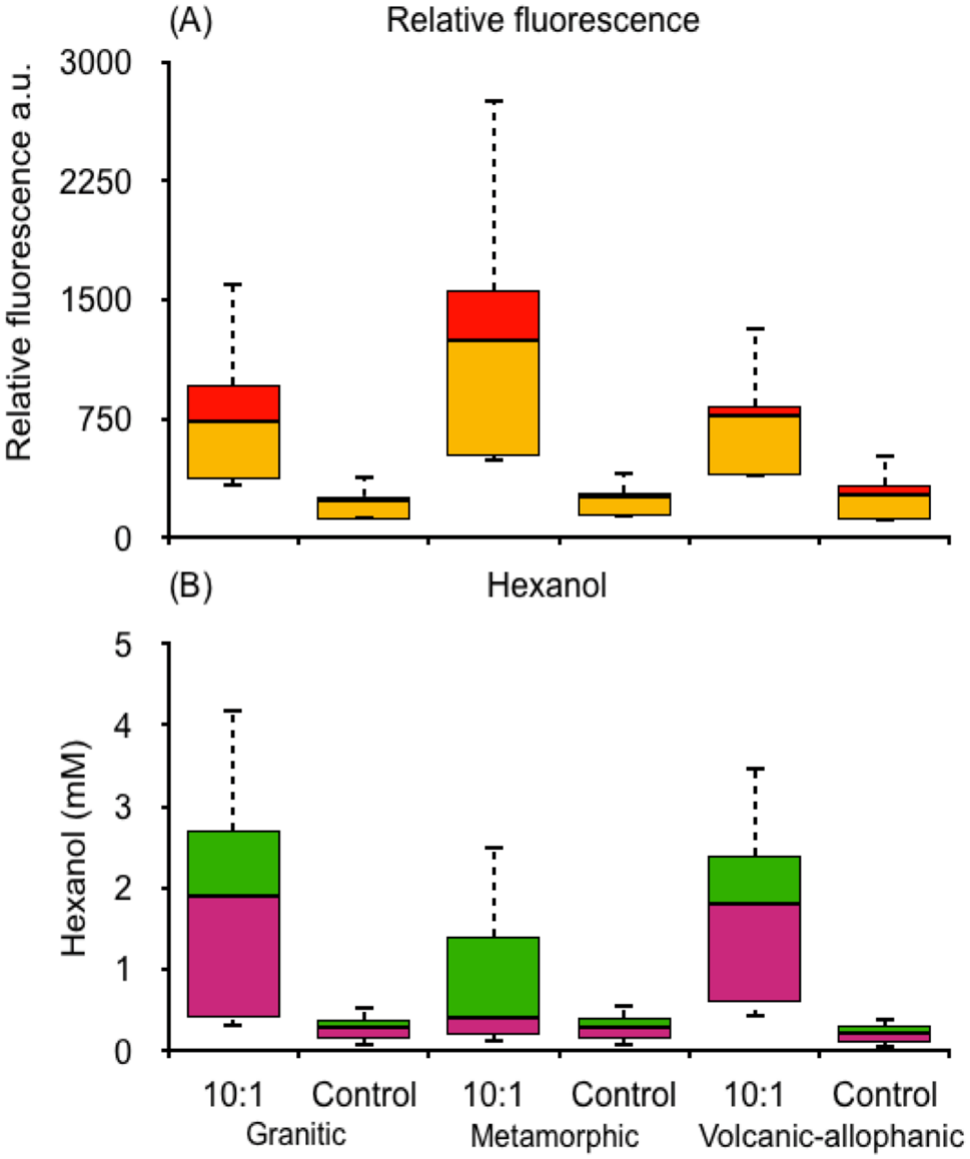
(**A**) Boxplot of relative fluorescence and (**B**) hexanol of free hydroxyl radicals from sterilized soils derived from granitic, metamorphic and volcanic-allophanic parent materials. The horizontal black lines and colours denote medians and quartiles, respectively.

A significant relationship was found between the relative fluorescence intensity and the concentration of hexanol oxidized (Fig. 6). The highest regression slope was found for Metamorphic soils (446.4 a.u. mM^−1^) (Fig. 6B), coinciding with the highest Fe levels found in this soil (Table 1). Maximal hydroxyl radical production at a H_2_O_2_:Fe(II) ratio of 10:1 was found in Metamorphic soils as observed from Confocal images (Fig. 7H). All soils exhibited high levels of relative fluorescence intensity compared to the control soils without H_2_O_2_ and Fe(II) additions (c.f. Fig. 7D–F with 7G–I).

## Discussion

Under aerobic incubation, approximately 31% of all CO_2_ released was abiotic and 69% was biotic. The lowest levels of organic C mineralization occurring during anaerobic and sterilized soil incubation correlate well with H_2_O_2_ consumption (Fig. 1). These results are attributed to Fenton reactions, since Fe(II)-HCl extractable were accumulated in soil and H_2_O_2_ was consumed to produce free radicals (Fig. 6). In the studied soils we found traces of H_2_O_2_ (Table 3) due to aeration and sample handling. We assumed that initial H_2_O_2_ in Anaerobic-abiotic treatment prior to incubation, triggered Fenton's reactions, which proceed in two steps. First, by oxidizing Fe(II)–Fe(III) at 76 L mol^−1^ s^−2^ to produce ·OH and second, reducing Fe(III) to Fe (II) at 0.01 L mol^−1^ s^−2^ to produce HO_2_^17^. Reduced and precipitated iron is continuously reused by Fenton reactions under anaerobic conditions and this should be the reason for similar amounts of iron found at the beginning and end of incubation.

On the other hand, the highest cumulative Fe(II) HCl-extractable were found in Anaerobic biotic incubations (non-sterilized soils). This can be explained due to the presence of obligated anaerobic Fe reducing bacteria, which were found in abundance in the same soils studied here (Merino et al. personal communication). Microorganism for methane oxidation involving iron reduction is also well documented^18^. Biotic factors, other than microorganisms, for example, ligninolytic enzymes activity can be preserved by clay minerals, even in sterilized soil^19^ (see discussion below). Abiotic mechanism, such as ferrous wheel, where SOM reduces Fe(III) to Fe(II), which is again oxidized in the presence of nitrate is also possible. This mechanism probably occurred in abiotic aerobic treatment^14^, that can explain the ferric iron release. Peroxidases enzymes activity could be well preserved in sterilized soils as well^19^.

Fenton reactions in combination with extracellular enzymes LiP and MnP mineralized eight times more CO_2_ than what was recorded in separated experiments (Fig. 3). These results are in line with the hypothesis that both Fenton reactions and enzyme activity involving MnP and LiP contribute considerably to SOC oxidation in temperate rainforest soils (Fig. 3). From the linear relationship found it can be determined that one mole of H_2_O_2_ yielded 0.5 mol of CO_2_ or that one mole of Fe(II) is required to consume 0.08 mol of H_2_O_2_ during Fenton + LiP + MnP treatment (Fig. 5). As previously indicated, this can be partially attributed to the potential preservation of enzyme activity in the soil matrix. This is an important and less widely considered mechanism where LiP and MnP are especially preserved in the clay minerals^7,8^. MnP and LiP remained active for extended periods of time, though they were sensitive to soil pH^20^. MnP and LiP activity in soil has been found to be 20–130 times higher than the activity of free enzymes in soil solution^8,21^. In our case, although the pH did not change throughout incubation, LiP was higher in Volcanic-allophanic soils (pH 5.1) and lower in Granitic soil (pH 3.6). In contrast, MnP activity increased as soil pH decreased (Fig. 4). Soil pH is an important regulator of enzyme stability, and this was notable in the case of LiP, which is less stable than MnP^20^. This mechanism can also explain the high potential oxidation capacity of the combined effects of Fenton reactions and enzymes on SOC mineralization. While LiP degrades lignin in soils with high pH levels, MnP does so in harsh environments with low pH levels. Our results are also supported by investigations of tropical soils, where Fe^2+^ involved in Fenton reactions and likely enzymatic activity substantially increased the rate of SOM decomposition^1,11,22^.

Direct evidence of Fenton reactions producing free radicals was also found from the relative fluorescence intensity and from the detection of free radicals by hexanol oxidation (Figs. 6, 7)^23,24^. The oxidation of DCFH_2_ by hydroxyl radical is irrefutable^25^. Free radical-like ·OH produced from Fe^2+^ and H_2_O_2_ added to soil can mediate the oxidation of DCFH_2_ through Fenton reactions. In the past it was believed that peroxidases similar to MnP and LiP interfered with DCFH_2_ oxidation. Now is clear that neither H_2_O_2_ nor Fe^2+^ is directly involved in DCFH_2_ oxidation while peroxidases and soluble Fe(II) may work as efficient catalysts^25^. Other oxidative mechanisms cannot be discarded. During anaerobic abiotic incubation, Fe(II) can react with nitrate present in soil solution, releasing CO_2_^14^. Several chemical substances can serve as alternative electron acceptors (e.g. sulphates, semiquinones) for the oxidation of SOC. These mechanisms can be thermodynamically favourable depending on reducing substrates available and soil pH levels^26^. Under anaerobic biotic conditions, iron reducing bacteria (IRB) can solubilize Fe(II) from Fe(III) releasing CO_2_ as well^27,28^.

In conclusion, when O_2_ is removed from soil, SOC is vulnerable to intense abiotic oxidation. This is likely the case because abiotic-mediated Fenton reactions in combination with exoenzymes LiP and MnP activities compete for SOC oxidation. Both are able to produce free radicals to oxidize lignin-like compounds. Radical ·OH reacts rapidly with nearly all organic molecules, either by abstracting hydrogens from aliphatic structures or by adding as an electrophile to aromatic ones^29^; thus, Fenton reactions and enzyme MnP and LiP interact through synergistic mechanisms to potentiate the oxidation of SOM^30^ in a wide variety of soils spanning different origins of varied parent materials and vegetation.

## Methods

### Study sites and sampling

Three soil types were selected (Table 2). The first was an Andisol^31^ (Volcanic–allophanic) formed from recent Volcanic ash deposited in the Andes. This was collected from a native temperate rainforest dominated by old growth *Nothofagus betuloides* (Mirb.) located in Puyehue National Park, where mean annual precipitation is typically > 8,000 mm year^−1^^12^. The soils are derived from basaltic scoria with high levels of allophane, imogolite, and ferrihydrite material^32^. The second soil type was an Ultisol (Metamorphic) sampled in Alerce Costero National Park in the coastal range. It is derived from Metamorphic-schist with high levels of illite-kaolinite^33^. Finally, an Inceptisol (Granitic) was selected from an ancient *Araucaria araucana* and *Nothofagus pumilio* forest in Nahuelbuta National Park. This soil type originates from intrusive Granitic rock parent materials. Mean annual precipitation in this ancient forest reaches > 1,491 mm and mean annual temperatures reach 13.3 °C^34^ (Table 2). From each soil sample, four composite soil samples were taken from the Ah mineral horizon (5–10 cm) after removing litter and organic horizons. The samples were then transported to a laboratory under cold conditions. All soils were homogenized and sieved (< 2 mm). Hereafter, all derived soils are identified as Granitic, Volcanic–allophanic and Metamorphic.

**Table 2:** Soil characteristics of study sites.

Soil^a^	Coord.^b^	Elev^c^ (m a.s.l.)	MAT^d^ (°C)	MAP^e^ (mm)	Veget.^f^	Soil order^g^
Granitic	37° 47′ S and 72° 59′ W	1,000	13.3	1,491	AA, NP	Inceptisol
Mathamorphic (mica-schists)	40° 12′ S and 73° 26′ W	1,048	9.5	4,000	DW, LP; NN, NP, PN, SC, WT	Ultisol
Volcanic–allophanic	40° 47′ S and 72° 12′ W	800	9.2	5,000	NB	Andisol

^a^Soil derived.

^b^Coordinates.

^c^Elevation.

^d^Mean annual temperature.

^e^Mean annual precipitation.

^f^Vegetation: AA, *Araucaria araucana*; DW, *Drimys winteri* J.R; LP, *Laureliopsis philippiana* (Looser) Schodde (Monimiaceae); NB, *Nothofagus betuloides* (Mirb); NN, *Nothofagus nitida* (Phil); NP, *Nothofagus pumulio*; PN, *Podocarpus nubigena* Lindl; SC, *Saxegothaea conspicua* (Lindl.) and WT, *Weinmannia trichosperma* Cav.

^g^Soil survey staff^18^.

### Analytical procedure

pH and electrical conductivity were directly measured in an aliquot of soil in a 1:2.5 suspension of soil:water. Soil organic C was determined using TOC-VCSH (Shimadzu, Kyoto, Japan), and total N was determined by Kjeldahl distillation (VELP, Usmate, Italy). For the determination of Fe (Fe_o_) and Mn (Mn_o_), 0.2 M ammonium oxalate at pH 3 was used^35^. Iron and Mn complexed with SOM (Fe_p_ and Mn_p_) were obtained using a solution of 0.1 M sodium pyrophosphate^36^. Dithionite-citrate-bicarbonate (Fe_d_) was used to identify exchangeable, crystalline, and complexed-SOM metals in the samples (see^36^ for further details). Fe and Mn concentrations were determined by atomic absorption spectroscopy (Perkin Elmer 3110, Waltham, Massachusetts, USA) conducted at 248.3 nm for Fe and at 279.5 nm for Mn using a nitrous oxide acetylene flame. Aluminium pyrophosphate (Al_p_), Al oxalate (Al_o_), and Si (Si_o_) were also determined as described above. Cation exchange capacity (CEC) and nutrient characterization were conducted as indicated by Sadzawka et al.^35^ (Table 3).

**Table 3:** Characteristics of soil^a^ used in the study.

Analysis	Units	Granitic	Methamorphic	Volcanic–allophanic
SOC^b^	%	9.20 ± 0.1	9.70 ± 0.2	11.40 ± 0.3
N total	%	0.47 ± 0.01	0.40 ± 0.00	0.60 ± 0.03
C:N ratio	Unitless	24.30	23.80	19.10
pH water	Unitless	3.60 ± 0.2	4.50 ± 0.2	5.1 ± 0.1
H_2_O_2_	µM g^−1^ soil	25.6 ± 0.7	28.0 ± 0.9	33.7 ± 0.5
LiP^c^	µg g^−1^ soil	1.23 ± 0.9	1.48 ± 0.1	2.31 ± 0.3
MnP^d^	µg g^−1^ soil	9.34 ± 1.1	8.12 ± 1.0	2.69 ± 0.0
Al_p_^e^	g kg^−1^ soil	0.70 ± 0.1	5.70 ± 0.02	11.0 ± 1.5
Al_o_^f^	g kg^−1^ soil	7 ± 0.02	0.73 ± 0.1	3.10 ± 0.2
Al_o_ + 1/2 Fe_o_	Unitless	1.25	1.85	3.80
Si_o_^g^	g kg^−1^ soil	2.20 ± 0.4	1.40 ± 0.1	3.10 ± 0.1
Al Saturation	%	80.00	93.50	22.40
Al exchangeable	cmol( +)kg^−1^ soil	10.92	18.41	1.19
Mn_o_	g kg^−1^ soil	0.02 ± 0.01	0.05 ± 0.00	0.09 ± 0.02
Mn_d_	g kg^−1^ soil	0.01 ± 0.01	0.01 ± 0.00	0.12 ± 0.03
Mn_p_	g kg^−1^ soil	0.001 ± 0.00	0.003 ± 0.00	0.027 ± 0.00
CEC_e_^h^	cmol( +)kg^−1^ soil	11.8	19.7	5.3
Clay type^i^		K	Q, I, K	Allophane-imogollite
Texture^j^		L	CL	SCL

^a^Derived from granitic, metamorphic, and volcanic-allophanic parent materials.

^b^Soil organic carbon.

^c^Lignin peroxidase.

^d^Manganese peroxidase.

^e^Pyrophosphate extractable Al.

^f^Oxalate extractable Al.

^g^Oxalate extractable Si.

^h^Effective cation exchange capacity.

^i^*Q* quartz, *K* kaolinite, *I* illite.

^j^*SCL* sandy clay loam, *CL* clay loam, *L* loam.

The total Fe concentration was determined from 100 mg of soil by adding 0.9 ml of 0.28 M hydroxylamine hydrochloride (2%) and 1 ml of 0.28 M HCl^26^. Approximately 100 μl of the extract was added to 4 ml of colour reagent (1 g ferrozine in a 6.5 M ammonium acetate solution). The amount of reduced iron Fe(II) (or Fe^2+^) was quantified from 0.1 g of soil added to 1 ml of 0.5 M HCl (now on Fe(II)-HCl)^34^ and shaken vigorously. Approximately 100 μl of this suspension was added to 4 ml of colour reagent (1 g ferrozine in 6.5 M ammonium acetate solution). The ferrozine complex standard was prepared with ferrous ethylene diammonium sulphate dissolved in 0.5 M HCl^37^. Fe^2+^ was determined spectrophotometrically at 562 nm (absorbance ferrozine-Fe^2+^ complex) after the colour developed. The concentration of oxidized Fe(III)-(oxyhydr)oxide was determined by calculating the difference between total Fe and Fe^2+^ concentrations (Table 1).

### Soil sterilization

Soils used for the incubation experiment (see below) were sterilized in an autoclave for 20 min at 121 °C for 3 consecutive days to remove the microbial population with resistant structures such as endospores and conidia. Furthermore, soils were fumigated with chloroform vapour in a vacuum chamber for 24 h^38^. Autoclaving was used because it does not create significant changes in the SOM structure, mainly leading to changes in carbohydrate and N‐alkyl regions of the ^13^C‐CP/MAS spectra due to the lysis of microorganisms and the subsequent loss of microbial C in the aqueous phase^39^. Gamma radiation was avoided because some reports indicate that it causes Fe reduction and oxidation^40–42^. Gamma radiation increased the bioavailability of Fe(III)(oxyhydr)oxide minerals, which resulted in increased Fe(III)(oxyhydr)oxide reduction^43^.

### Microorganism culture and enzyme extraction

White rot fungi were isolated from wood logs in the same areas where soil sampling was conducted. The white rot fungi were cultured and maintained in Koroljova–Skorobogat’ko medium at pH 5^44^. This is a typical medium used to grow fungi for enzymatic extraction when required^45,46^. The medium (g l^−1^) consisted of: 3.0 peptone, 10.0 glucose, 0.6 KH_2_PO_4_, 0.001 ZnSO_4_, 0.4 KH_2_PO_4_, 0.0005 FeSO_4_, 0,05 MnSO_4_, and 0,5 MgSO_4_ and the fungi exhibited strong growth^45,46^. Sterile Koroljova–Skorobogat’ko medium was dispersed into sterile 250 ml Erlenmeyer flasks at a rate of 50 ml of medium per flask. The flasks were inoculated with homogenized mycelia suspension and incubated in an orbital shaker at 30 °C and at a rate of 200 rpm. The flasks with growing cultures of white root fungi were removed at different time intervals over the course of the experiment for processing. MnP and LiP were isolated from fungal culture slants in a Koroljova liquid broth medium (10 ml) after 24, 48, 72, 96 and 120 h to obtain mycelia and spores by centrifugation at 4 °C (10,000 rpm for 10 min). All purification and protein concentration steps were performed at 4 °C. Small pre-weighed quantities of ammonium sulphate were added to 250 ml of culture supernatant from 20 to 80% saturation. Each precipitated fraction was separated by centrifugation at 10,000 rpm for 15 min at 4 °C, dissolved in a minimum volume of 0.1 M Tris–HCl (pH 9.0) and dialyzed twice for 6–8 h against the same buffer. The dissolved fractions were stored at 4 °C. Ligninolytic enzyme activity (LiP and MnP) was estimated in the crude culture filtrate and ammonium sulphate precipitates using the standard protocols described below.

### Enzyme assay

MnP activity was measured by the oxidation of MnSO_4_ (1.0 mM) substrate. Reactions were conducted in a 3 ml cuvette containing 2.5 ml of buffer (20 mM sodium tartrate at pH 4.5), 1.0 ml of substrate, 1.0 ml of enzyme extract from the supernatant, and 0.5 ml of 2.0 mM H_2_O_2_. Manganese peroxidase was determined spectrophotometrically at 238 nm^47^. Lignin peroxidase was evaluated using veratryl alcohol (2.0 mM) as a substrate. The reactions were carried out in 3 ml cuvettes containing 1.25 ml of 50 mM sodium tartrate buffer at pH 2.5, 0.5 ml of enzyme extract, 0.25 ml of substrate, and 0.5 ml of 500 μM H_2_O_2_. Lignin peroxidase was measured at 310 nm. All oxidative enzymatic activities were expressed as units (U) per millilitre (i.e., one millimole of substrate oxidized per minute)^48^.

The standard Bradford method was used to estimate the concentration of proteins in the supernatant. Bovine serum albumin (BSA) was used as a standard. One millilitre of enzyme extract was mixed with 1 ml of Bradford reagent (Amresco, USA) and incubated for five days. Protein concentrations were measured at 595 nm. Approximately 3 ml of mixed enzyme extract was used for the identification of MnP and LiP. We used Type II horseradish peroxidase (Sigma Aldrich) dissolved in phosphate buffer as an enzyme standard. HPLC–MS/MS (GE Healthcare, USA) was used for protein separation. The outflow was monitored at 280 nm for protein detection. The fractions were assayed for MnP and LiP activity and total protein content.

### Experiment 1: incubation under biotic and abiotic conditions

Four replicates of 13 g of moist (80% of field capacity) sterilized soil (abiotic) and another portion of non-sterilized soil (biotic) were incubated in serum bottles (120 mml) at 20 °C in anaerobic conditions. Another round of abiotic and biotic incubation was conducted under aerobic conditions. Serum bottles were equipped with a septum for gas sampling. Anaerobic incubations were previously tested by injecting oxygen-free gas (N_2_) into the headspace of each serum bottle until < 2% O_2_ was reached (PCE Instrument model PCE-228-R, Germany)^49^. Anaerobic conditions were monitored and CO_2_ was collected after 0.3, 4, 8, 12, 24 and 36 h of incubation. Preliminary studies indicate that after further incubation for > 3 days, CO_2_ levels increased little. For gas sampling, 10 ml was extracted using a syringe, and this was then injected into a gas chromatograph coupled with thermal conductivity and a flame photometric detector.

### Experiment 2: induced fenton reactions

Fenton reactions were induced by adding various hydrogen peroxide (H_2_O_2_) and one Fe(II) concentration to all sterilized soils in ratios of 5:1, 10:1, and 20:1^17,50,51^ by adding 120–143 ml, 0.1 M H_2_O_2_ and 1.29 g Fe^2+^ kg^−1^ soil as FeCl_2_ (Sigma Aldrich, USA).

### Experiment 3: LiP and MnP activity

Sterilized soils were inoculated with peroxidase enzymes and H_2_O_2_. Iron (II) was not added in this experiment because MnP and LiP do not require free Fe. Enzymatic activity in the soils was monitored for 36 h spectrophotometrically at 310 nm for LiP and at 238 nm for MnP using a Tecan Infinite 200 PRO spectrophotometer (Durham, NC). The Initial activity of both enzymes was determined to each soils previously to the sterilization. Detailed values of LiP (1.23 ± 0.9–2.31 ± 0.3 µg g^−1^ soil) and MnP (2.69 ± 0.0–9.34 ± 1.1 µg g^−1^ soil) are presented in Table 3. This experiment is referred to as Peroxidase.

### Experiment 4: combined fenton reactions and ligninolitic enzymes LiP and MnP

One millilitre of inoculum from a combined extract of LiP and MnP was added to sterilized soils with the addition of H_2_O_2_ and Fe(II) to induce Fenton reactions as described above. The soils were incubated under anaerobic conditions (in Fenton + LiP + MnP).

### Hydrogen peroxide and Fe(II)-HCl determination

Experiments 1–4 were performed in parallel for destructive sampling to monitor Fe(II)-HCl solubilization and hydrogen peroxide consumption. Hydrogen peroxide consumption was determined using the iodometric titration method^52^. This method measures the concentration (mg l^−1^) of an oxidizing agent in solution. While it is somewhat less accurate than permanganate titration, it is less susceptible to interferences by SOM. The method has been applied to plant tissues^53,54^ and soils^55^. In brief, at each sampling time soil samples were frozen to − 18 °C to stop enzymatic activity. Then, 150 mg of frozen samples were homogenized with 1 ml of solution containing 0.25 ml of 0.1% trichloroacetic acid, 0.5 ml of 1 M KI, and 0.25 ml of 10 mM potassium phosphate buffer. The homogenized suspension was incubated at 4 °C for 10 min. H_2_O_2_ content were monitored spectrophotometrically at 390 nm and final values are expressed in μmol g^−1 ^of fresh weight soil^48^. H_2_O_2_ consumption (%) was estimated as:

1 $$ {\text{H}}_{2} {\text{O}}_{2} {\text{consumption }} = \left( {\frac{{{\text{H}}_{2} {\text{O}}_{2} {\text{added}} - {\text{H}}_{2} {\text{O}}_{2} {\text{remaining}}}}{{{\text{H}}_{2} {\text{O}}_{2} {\text{added}}}}} \right){*}100 $$

The initial H_2_O_2_ content from each soils were determined previously to sterilization and ranged between 25.6 ± 0.7 and 33.7 ± 0.5 µM g^−1^ soil (Table 3).

### Free radical detection

The presence of hydroxyl radicals in the soil was first tested using hexanol substrate. Approximately, 5 g of each sterilized soil (13 replicates) was incubated for 36 h in anaerobic conditions with 5 ml of hexanol (2 mM) (Sigma-Aldrich). A H_2_O_2_:Fe(II) ratio of 10:1 was used to induce Fenton reactions (see below). Hydroxyl radicals do not react strongly with the superoxide anion but with hexanol, as hydroxyl radicals oxidize preferentially short-range organic molecules^23,24^. In total, 39 soil samples were analysed for 10 randomly selected regions of interest (ROI, 33,489 μ^2^). Hexanol oxidation was calculated as the difference between the initial amount and the amount of hexanol remaining. The hexanol was quantified using a Hewlett-Packard 5890A gas chromatograph (Thermo Fisher, Waltham, Massachusetts USA) with a flame ionization detector equipped with a 15 m × 0.53 mm DB-1 capillary column.

In the fluorescence experiment, the generation of ·OH radicals was examined in anaerobic and sterilized soils using a 2′,7′-dichlorodihydrofluorescein diacetate (DCFH_2_)^25^ fluorescent probe in an excitation/emission of 488/530 nm and via laser scanning confocal microscopy (CLSM) (Olympus Fluoview 1000, Florida, USA). Maximum fluorescence emissions were found for a ratio of 10:1 H_2_O_2_:Fe(II). Using a closed vacuum plate with 50 μl of the fluorescent DCFH_2_ probe^56^, the samples were analysed for free radicals after 36 h of incubation^57^. The emissions observed using CLSM were attributed to hydroxyl radicals reacting in the soil. The images were processed using image processing software (software FV10-ASW v.0.2c), and fluorescence intensity was expressed as relative fluorescence (AU) as given by the software. A control soil without H_2_O_2_ and Fe(II) additions was also included.

### Statistical analysis

Two-way ANOVAs were conducted to determine significant differences in CO_2_ released, hydrogen peroxide consumed, and Fe(II)-HCl solubilized for experiment 1. To test differences for the same variables in the second, third, and fourth experiments, one-way ANOVAs were performed. Regressions between CO_2_ and H_2_O_2_ or Fe(II)-HCl extractable and between H_2_O_2_ and Fe(II)-HCl extractable were performed. All analyses were conducted using XLSTAT software by Addinsoft (Premium) 2019, version 4.1. Significant differences were set at a *p* value of 0.05. Datasets were tested for normal distributions and homoscedasticity. Datasets abnormally distributed were log transformed when necessary.

## Supplementary information

Supplementary file1 (DOCX 29 kb)
