# Supplementary material for: Synergy effect of peroxidase enzymes and Fenton reactions greatly increase the anaerobic oxidation of soil organic matter
Source: Sci Rep. 2020 Jul 9;10:11289. doi: 10.1038/s41598-020-67953-z (PMC7347925; doi:10.1038/s41598-020-67953-z)
Supplement: Supplementary file 1 — Supplementary file1 (DOCX 29 kb) [file 41598_2020_67953_MOESM1_ESM.docx]

**Synergy effect of peroxidases enzymes and Fenton reactions greatly increase the anaerobic oxidation of soil organic matter**

Carolina Merino, Yakov Kuzyakov, Karina Godoy, Pablo Cornejo, Francisco Matus*

^*^Corresponding author: E-mail address: francisco.matus@ufrontera.cl (F. Matus)

**Table S1.**Two ways ANOVA test for the total CO_2_ evolved, H_2_O_2_ consumption and Fe(II)-HCl solubilization from aerobic and anaerobic sterilized (abiotic) and non-sterilized (biotic) treated soils derived from granitic, metamorphic and volcanic-allophanic parent materials, incubated at 20 ºC for 36 hours on aerobic and anaerobic biotic and abiotic each (treatments).

|  | DF | Sum of Squares | | F value | *p* >F |
| --- | --- | --- | --- | --- | --- |
| CO_2_ | | |  | | |
| Soil (A) | 2 | 48205 | | 35.983 | < 0.001 |
| Treatment (B) | 3 | 145545 | | 72.429 | < 0.001 |
| A x B | 6 | 21128 | | 5.257 | < 0.001 |
| H_2_O_2_ consumption |  |  | |  |  |
| Soil (A) | 2 | 609.9 | | 7.4642 | < 0.001 |
| Treatment (B) | 3 | 6296.1 | | 51.3676 | < 0.001 |
| A x B | 6 | 338.1 | | 1.3793 | 0.22 |
| Fe(II)-HCL |  |  | |  |  |
| Soil (A) | 2 | 2.469 | | 1.3009 | 0.27 |
| Treatment (B) | 3 | 154.035 | | 54.1004 | < 0.001 |
| A x B | 6 | 20.342 | | 3.5722 | 0.002 |

**Table S2.** Two ways ANOVA test for the total CO_2_ evolved from soils derived from granitic, metamorphic and volcanic-allophanic parent materials as affected by Fenton reaction induced by the addition of H_2_O_2_ and Fe(II) at different ratios (5:1; 10:1; 20:1), the activity of enzymes manganese peroxidase (MnP) and lignin peroxidase (LiP) and the combined effect of Fenton+MnP+LiP after 36 hours of incubation at 20 ºC.

|  | DF | Sum of Squares | | F Ratio | *p* >F |  |
| --- | --- | --- | --- | --- | --- | --- |
|  | | | | | |  |
| Fenton reaction | | |  | | |  |
| Soil (A) | 2 | 0.0716 | | 3.8264 | 0.0331 |  |
| Ratio (B) | 1 | 0.0024 | | 0.2517 | 0.6195 |  |
| A x B | 2 | 0.0062 | | 0.3294 | 0.7219 |  |
|  | | | | | |  |
| MnP and LiP | | |  | | |  |
| Soil (A) | 2 | 61.7290 | | 10.2474 | < 0.001 |  |
| Ratio (B) | 1 | 13.6806 | | 4.5421 | 0.0414 |  |
| A x B | 2 | 4.8252 | | 0.801 | 0.4582 |  |
|  | | | | | |  |
| Fenton+MnP+LiP | |  | |  |  |  |
| Soil (A) | 2 | 0.0218 | | 3.4532 | 0.0447 |  |
| Ratio (B) | 1 | 0.1039 | | 32.9579 | <.0001 |  |
| A x B | 2 | 0.0746 | | 11.828 | 0.0002 |  |

**Table S3.** Relationship between CO_2_ (mg kg^-1^) evolved and H_2_O_2_ consumption (%), CO_2_ and Fe(II)-HCl (mg kg^-1^) in soils derived from granitic, metamorphic and volcanic allophanic parents materials . All regressions were significant at *p* < 0.01.

| Treatment^a^ | Soil | CO_2_ (y) vs H_2_O_2_(x) | CO_2_ (y) vs Fe(II)-HCl(x) | H_2_O_2_(y) vs Fe(II)-HCl(x) |
| --- | --- | --- | --- | --- |
| Fenton | Granitic | y = 0.25x+1.58  R^2^ = 0.95 | y = 1.57x+0.46  R^2^ = 0.97 | y = 6.01x-3.39  R^2^ = 0.96 |
|  | Metamorphic | y = 0.42x-10.7  R^2^ = 0.93 | y = 10.4x-13.9  R^2^ = 0.87 | y = 24.4x-6.78  R^2^ = 0.91 |
|  | Volcanic-  allophanic | y = 0.49x-1.39  R^2^ = 0.98 | y = 3.77x-5.84  R^2^ = 0.86 | y = 7.75x-9.26  R^2^ = 0.89 |
| MnP+LiP | Granitic | y = 0.47x+0.28  R^2^ = 0.97 | y = 3.76x+1.42  R^2^ = 0.89 | y = 7.72x+2.59  R^2^ = 0.88 |
|  | Metamorphic | y = 0.50x-0.97  R^2^ = 0.97 | y = 5.53x+0.95 R^2^ = 0.96 | y = 10.8x+6.85 R^2^ = 0.93 |
|  | Volcanic-  allophanic | y = 0.35x-3.58  R^2^ = 0.99 | y = 4.60x+1.34  R^2^ = 0.95 | y = 12.9x+13.9  R^2^ = 0.95 |
| Fenton+MmP+LiP | Granitic | y = 2.32x-29.4  R^2^ = 0.97 | y = 24.4x-14.0  R^2^ = 0.90 | y = 10.4x+6.91  R^2^ = 0.91 |
|  | Metamorphic | y = 4.9x-57.8  R^2^ = 0.98 | y = 64.9x-65.5 R^2^ = 0.92 | y = 13.1x-1.2 R^2^ = 0.92 |
|  | Volcanic-  allophanic | y = 2.5x-23.6  R^2^ = 0.96 | y = 19.7x-22.6  R^2^ = 0.95 | y = 7.51x+1.51  R^2^ = 0.92 |

^a^See Table S2 for treatments description.
